# Hydronephrotic Urine in the Obstructed Kidney Promotes Urothelial Carcinoma Cell Proliferation, Migration, Invasion through the Activation of mTORC2-AKT and ERK Signaling Pathways

**DOI:** 10.1371/journal.pone.0074300

**Published:** 2013-09-04

**Authors:** Chi-Hao Chang, Jian-Ri Li, Kuo-Hsiung Shu, Yun-Ching Fu, Ming-Ju Wu

**Affiliations:** 1 Institute of Clinical Medicine, National Yang Ming University, Taipei, Taiwan; 2 Department of Surgery, Division of Urology, Taichung Veterans General Hospital, Taichung, Taiwan; 3 Department of Medicine, Division of Nephrology, Taichung Veterans General Hospital, Taichung, Taiwan; 4 Department of Pediatrics, Taichung Veterans General Hospital, Taichung, Taiwan; 5 School of Medicine, Chung-Shan Medical University, Taichung, Taiwan; 6 Graduate Institute of Clinical Medical Science, School of Medicine, China Medical University, Taichung, Taiwan; 7 Institute of Biomedical Science, National Chung Hsing University, Taichung, Taiwan; University of Central Florida, United States of America

## Abstract

Obstructive nephropathy is the most common presentation of urothelial carcinoma. The role of the urine in the obstructed kidney namely “hydronephrotic urine” in urothelial carcinoma has not been extensively explored. This study aims to evaluate whether hydronephrotic urine in the obstructed kidney could promote urothelial carcinoma. The hydronephrotic urine was collected from the obstructed kidneys of Sprague-Dawley rats induced by different periods of unilateral ureteral obstruction (UUO). By the inhibition of LY294002 and PD184352, we confirm that hydronephrotic urine promotes urothelial carcinoma cell (T24) and immortalized normal urothelial cells (E6) proliferation, migration and invasion in a dose-dependent manner through the activation of the mTORC2-AKT and ERK signaling pathways. Hydronephrotic urine also increases the expression of cyclin-D2, cyclin-B and CDK2. It also decreases the expression of p27 and p21 in both urothelial carcinoma cells and normal urothelial cells. By the protein array study, we demonstrate that many growth factors which promote tumor cell survival and metastasis are over-expressed in a time-dependent manner in the hydronephrotic urine, including beta-FGF, IFN-γ, PDGF-BB, PIGF, TGF-β, VEGF-A, VEGF-D and EGF. These results suggest that hydronephrotic urine promotes normal and malignant urothelial cells proliferation, migration and invasion, through the activation of the mTORC2-AKT and ERK signaling pathways. Further investigation using live animal models of tumor growth may be needed to clarify aspects of these statements.

## Introduction

Urothelial carcinoma is the most common type of malignancy in both long-term dialysis patients and kidney transplant recipients in Taiwan [1–3]. Hydronephrosis, determined by ultrasonography, is the specific common finding of urothelial carcinoma in these patients [2–4]. Although the obstruction of urinary tract by the tumorous lesion is a reasonable cause of hydronephrosis, cancerous lesions are not always present in the obstructive site [2,3]. Moreover, a preoperative hydronephrosis grade can independently predict worse pathological outcomes in patients undergoing nephroureterectomy for upper tract urothelial carcinoma [4]. Therefore, we suggest that the “hydronephrotic urine” namely the urine in the obstructed kidney may play an important role to promote the growth and progression of urothelial carcinoma.

Any obstruction which occurs along the urinary tract may lead to an increased pressure within the structures of the kidney due to the inability of urine to pass from the kidney to the bladder. The distension and dilation of the renal pelvis calyces is so-called “hydronephrosis”. Hydronephrosis is the result of several abnormal pathophysiological occurrences. Congenital structural abnormalities of urinary tract, urolithiasis, urothelial carcinoma, and injury to the urinary tract related to infection, surgery, or radiation therapy could lead to hydronephrosis. All of these factors could lead to the destruction of the delicate tissues that make up the filtration system within the kidneys, which might eventually result in infection, stone formation, tubulointerstitial fibrosis or loss of renal function [5–8].

In the rat model of hydronephrosis in this study, kidney obstruction was induced by unilateral ureteral ligation [9,10]. Unilateral ureteral obstruction causes renal inflammation and leads to interstitial mononuclear cells infiltration, marked tubular dilatation, and tubular cells apoptosis or necrosis [9]. Many growth factors and cytokines were overexpressed after ureteral obstruction, including platelet-derived growth factor (PDGF), transforming growth factor-beta (TGF-β), insulin-like growth factor-I (IGF-I), Interleukin-6 (IL-6) [7,8,10,11]. Besides, the expression of several proto-oncogene proteins such as c-fos, c-jun, c-myc and Ras were also increased after ureteral obstruction [6,11]. Although it is reasonable to predict the overexpression of the growth factors and cytokines in the hydronephrotic urine, it is not fully clarified.

The mammalian target of rapamycin (mTOR) is a serine/threonine protein kinase that plays a critical role in many growth factor receptors downstream networks [12–17]. Two major regulators of the mTOR complexes (mTORC) signaling pathway are mTORC1 and mTORC2. The mTORC1 is rapamycin-sensitive and contains mTOR, raptor and mLST8, while mTORC2 contains mTOR, rictor, mLST8 and sin1 (stress-activated protein kinase-interacting protein). The mTORC1 regulates mRNA translation and ribosome biogenesis, whereas mTORC2 plays an important role in the phosphorylation and subsequent activation of AKT [18–22]. Recently, we demonstrated that the rictor-dependent AKT activation in rat model of urothelial carcinoma could be a consequence of mTORC1 inhibition [23]. The growth factors dependent activation of ERK and mTORC2-AKT signaling pathway play an important role in the pathogenesis of human cancers [24–26]. Thus, we were interested in whether the growth factors in the hydronephrotic urine of obstructed kidney could activate mTORC and consequently promote urothelial carcinoma cells proliferation, survival and migration.

At present, the indication of the aggressive relief of hydronephrosis or preventive nephrectomy for patients with severe hydronephrosis is not conclusive even for the non-functional kidneys [27,28]. In Taiwan, some patients with hydronephrosis eventually develop urothelial carcinoma. Our results could provide more evidence that may assist physicians in the selection of aggressive treatment strategies for patients with severe hydronephrosis.

## Materials and Methods

### Animal model and the collection of urine samples

The rat model of obstructive nephropathy was induced by unilateral ureteral obstruction (UUO). Male Sprague-Dawley rats were obtained from the National Laboratory Animals Center (Taipei, Taiwan). Rats were sacrificed after 1, 2, 3, 4, and 5 weeks of UUO according the planned schedule. Samples of hydronephrotic urine from the obstructed kidneys were collected in 10 c.c. syringes and then filtered through 0.22µm filters and stored at -20°C or below. Samples of urine from rats with or without UUO were collected by metabolic cage for 12 hrs. All experimental procedures were performed according to the animal care and ethics legislation. The protocol has been approved by the Animal Care and Research Committee of Taichung Veterans General Hospital (permit numbers: LA-100814).

### Cell culture, Hydronephrotic urine, Epidermal Growth Factor and Inhibitor treatment

The human transitional cell carcinoma cell line T24 (G3, p53 mutant type) was obtained from American Type Culture Collection (Rockville, MD) and the normal human urothelial cell line E6 was obtained from the laboratory of Dr. Hong-Chen Chen (Department of life science, National Chung-Hsing University, Taichung, Taiwan) and Dr. Hsiao-Sheng Liu (Department of Microbiology & Immunology, National Cheng Kung University, Tainan, Taiwan) [29]. The T24 cells were maintained in McCoy’s 5A medium and the E6 cells were maintained in Dulbecco’s modified Eagle’s medium (Sigma-Aldrich, St. Louis, MO) containing 10% fetal bovine serum supplemented with 100 U/ml penicillin-G, 100 g/ml streptomycin, and 2 mM L-glutamine (CCS; HyClone, Logan, UT).

The T24 cells and E6 cells were cultured in serum-free medium for 18h with or without hydronephrotic urine. After treatment the cells subjected to analysis by cell functions and protein function assays. The T24 cells and E6 cells were stimulated with EGF (100ng/ml) and T24 cells and E6 cells were also treated with LY294002 (20µM) (GIBCO) and PD184352 (10µM) (BioVision) to inhibit mTORC2-AKT and ERK pathway.

### Wound healing assay for cell migration activity

T24 cells and E6 cells (2x10^4^) were seeded onto 6-well tissue culture dishes and cultured in the medium containing 10% FBS to a confluent cell monolayer, then carefully wounded using sterile 1-ml pipette tips. All cell debris was removed with PBS. The cells were then incubated in serum-free medium with or without hydronephrotic urine for 24 hrs and photographed under a phase contrast microscope. Experiments were repeated in triplicate.

### Transwell motility assay for cell invasion activity

T24 cells and E6 cells were seeded at 10^5^ in 6-cm dishes for 12 hrs and then changed the medium containing 5% FBS and hydronephrotic urine from 1–5 weeks of UUO for 12 hrs. Next, T24 cells and E6 cells (2x10^4^) were plated in the upper compartment of an 8-µm pore-size transwell migration chamber (Corning, Acton, MA) which was coated growth factor reduced matrigel (Millipore) and cultured in serum-free medium for 24 hrs. The cells on the upper surface were then removed by wiping with a cotton swab, after which the filter gently removed from the chamber, and the cells were mounted on glass slides. The cells that had invaded the filter and attached to its lower surface were fixed, stained with Giemsa stain solution (Sigma), and counted in all the microscopic fields (at 100 X magnifications) per filter. Experiments were repeated in triplicate.

### Cell viability analysis

Cell viability analysis was performed with trypan blue staining to confirm the cell survival and the numbers of live cells were counted with hemocytometer. T24 cells and E6 cells were cultured and treated with or without hydronephrotic urine for 0 hrs, 24 hrs, and 48 hrs. The cells were digested with 0.05% trypsin and the cell suspension was collected. The numbers of live cells were counted with hemocytometer under microscopy.

### Protein array assay

The protein array assay was performed with the RayBio Human Angiogenesis Antibody Array C Series 1000 (RayBiotech, Inc., Norcross, GA) to identify the growth factors and cytokines in the normal urine and hydronephrotic urine. The normal urine samples collected from the normal (non-UUO) rats and the hydronephrotic urine collected from the rat kidneys after one to three weeks of UUO were incubated with Human Angiogenesis Antibody Array membranes, following the protocol and reagents that RayBio provided to analyze the samples. Densitometry and statistical analysis were performed using the same methods as for the immunoblots. The results were normalized to the positive and negative controls on the array. The Multi Gauge V 3.0 (Fuji LAS-3000 luminescence image system) software was used for the analysis of immunoblots signals.

### Western blot analysis

For Western blots analysis, cells and tissues were prepared with primary antibodies, as previously reported. Primary antibody was detected using horseradish peroxidase-linked goat anti-mouse or goat anti-rabbit IgG antibody at a 1:1,000 dilution (Santa Cruz) and visualized with SuperSignal West Pico chemiluminescent substrate (Thermo Scientific, Rockford, IL). Primary antibodies used in this study included mTOR-2481p, ERK-p, cyclin D1/2 (Millipore), mTOR, AKT, ERK, cyclin E, cyclin A, cyclin B, CDK2, CDK4, CDK6, p21 (Cell signaling), AKT Ser473 (Santa Cruz), p27 (BD biosciences), alpha-tubulin and beta-actin (CHEMICON).

### Morphometric analysis

After fixing the kidney in 10% formalin, the lumen was inspected for grossly visible lesions. All immunohistochemical studies were performed on paraffin-embedded sections. The 4-µm-thick deparaffinized sections were incubated with the primary antibodies of phosphor-mTOR (Ser2481, 1:50, abcam) and phosphor-AKT (Ser473, 1:50, Santa Cruz Biotechnology Inc., Santa Cruz, CA). As a negative control, the primary antibody was replaced with normal rabbit IgG, without staining.

### Statistics

All data are expressed as mean ± Standard Deviation. All statistical analyses were performed using the Statistical Package for the Social Sciences (SPSS version 13.0 for Windows, SSPS Inc., Chicago, IL). Student’s t-test was used to determine whether there was a significant difference between two means. Statistical significance was defined as P value less than 0.05.

## Results

### Hydronephrotic urine promoted urothelial cell viability

**Figure 1:**
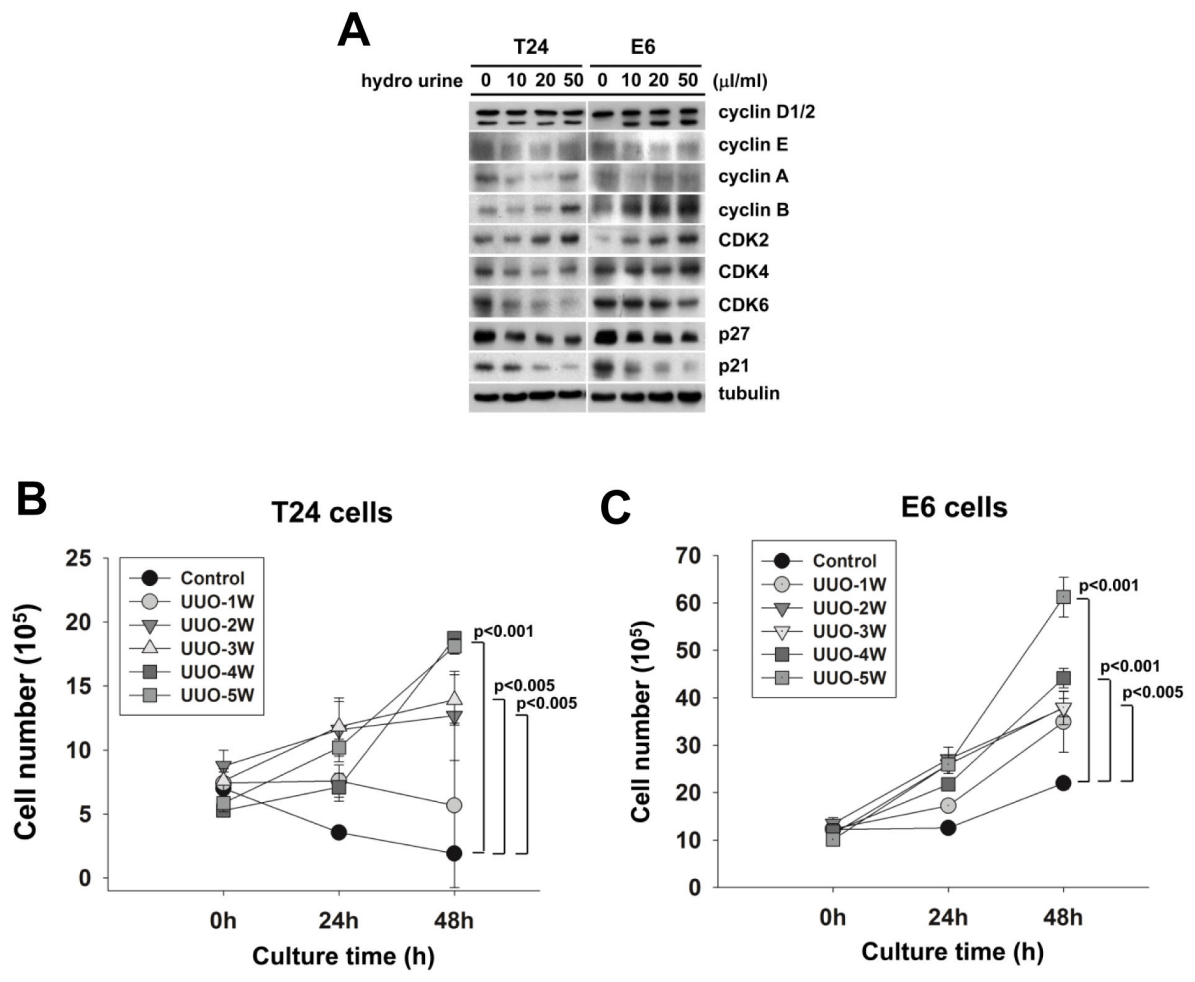
Hydronephrotic urine induced the expression of the cell cycle-regulated proteins and promoted the proliferation of T24 cells and E6 cells. (A) T24 cells and E6 cells were cultured in the hydronephrotic urine (10, 20, 50 µl/ml) at 3 weeks after UUO following serum starvation for 24 hrs. The expressions of cell cycle regulated proteins were analyzed by western blotting. (B) T24 cells were cultured in the hydronephrotic urine from 1, 2, 3, 4 and 5 weeks after UUO under serum starvation. Hydronephrotic urine at 2, 3, 4 and 5 weeks after UUO promoted cell proliferation but hydronephrotic urine did not promote T24 cells proliferation at 1 week after UUO (Control: without hydronephrotic urine treatment). (C) E6 cells were cultured in the hydronephrotic urine from 1, 2, 3, 4 and 5 weeks after UUO. Hydronephrotic urine at 1 to 5 weeks after UUO promoted proliferation of E6 cells (Control: without hydronephrotic urine treatment).

The hydronephrotic urine treatment after 3 weeks of UUO decreased the expression of p27 and p21 and increased the expression of cyclin B and CDK2 in both T24 cells and E6 cells but only increased the expression of cyclin-D2 in E6 cells (Figure 1A). This result indicated that hydronephrotic urine promoted the proliferation by the inhibition of p27, p21 and overexpression of cyclin-D2, cyclin-B and CDK2.

The hydronephrotic urine treatment after 2-5 weeks of UUO, but not hydronephrotic urine treatment after 1 week of UUO, increased the viability of T24 cells (Figure 1B). All urine samples which were collected from obstructed kidneys after 1-5 weeks UUO increased the cell viability of E6 cells (Figure 1C).

### Hydronephrotic urine promoted T24 cells and E6 cells migration and invasion

**Figure 2:**
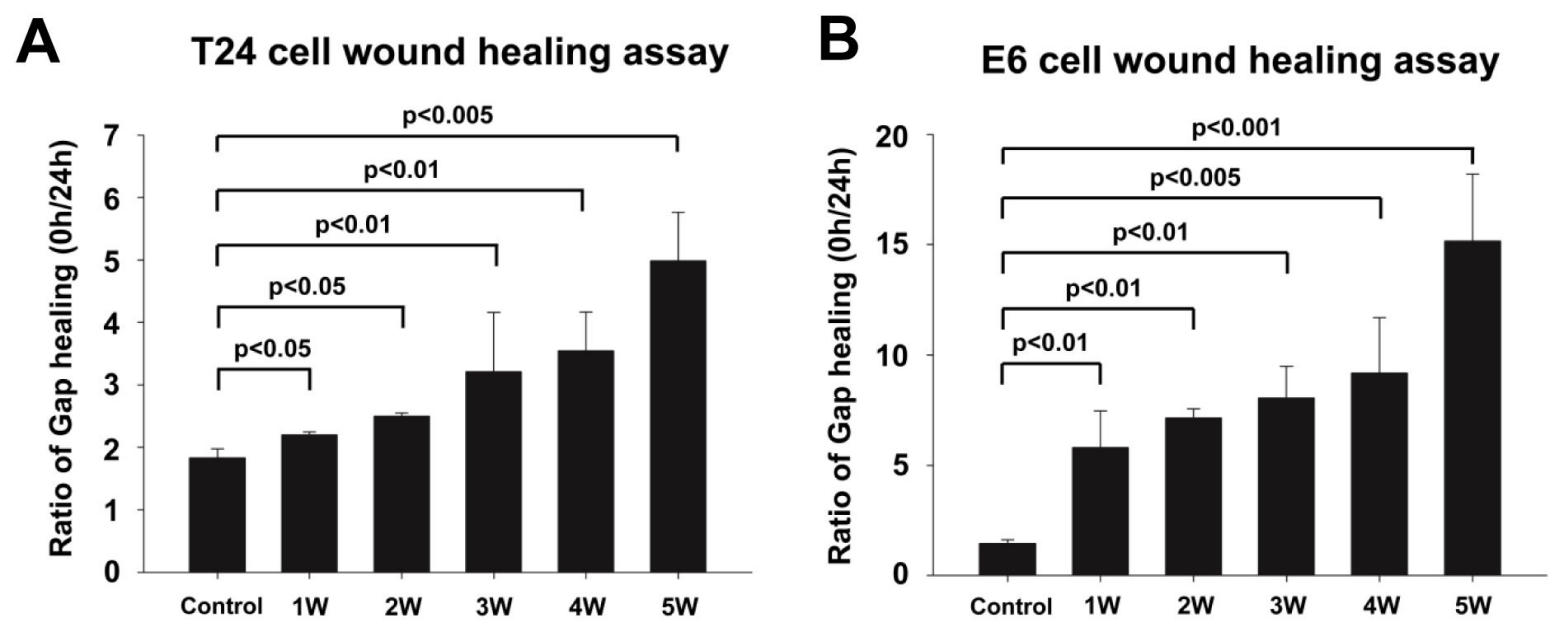
Hydronephrotic urine promoted the migratory capability of T24 cells and E6 cells. A wound healing assay was used to assess the migratory capability of T24 cells and E6 cells. (A, B) T24 cells and E6 cells were cultured in hydronephrotic urine from 1 to 5 weeks after UUO for 24 hrs. The ratio of gap healing was defined as the gap distance at 0h divided by the gap distance at 24h (0h/24h) (Control: without hydronephrotic urine treatment).

A wound healing assay was used to assess the migratory capability of T24 cells and E6 cells. The hydronephrotic urine significantly promoted the cell migration in both T24 cells and E6 cells. Figure 2 showed that the hydronephrotic urine collected after a longer duration of UUO was associated with larger ratio of gap distance (0h/24h) in both T24 and E6 cells. In T24 cells, compared to control group (without hydronephrotic urine treatment), the ratio of gap distance for hydronephrotic urine treatment from 1–5 weeks obstructed kidney induced by UUO increased to 1.2 fold, 1.4 fold, 1.8 fold, 1.9 fold and 2.7 fold, respectively (p<0.05) (Figure 2A). In E6 cells, the ratio of gap distance of hydronephrotic urine treatment after 1-5 weeks of UUO also significantly increased to 4 fold, 4.9 fold, 5.5 fold, 6.3 fold and 10.4 fold, respectively (p<0.05) (Figure 2B). These results indicate that hydronephrotic urine promotes migration of T24 and E6 cells.

**Figure 3:**
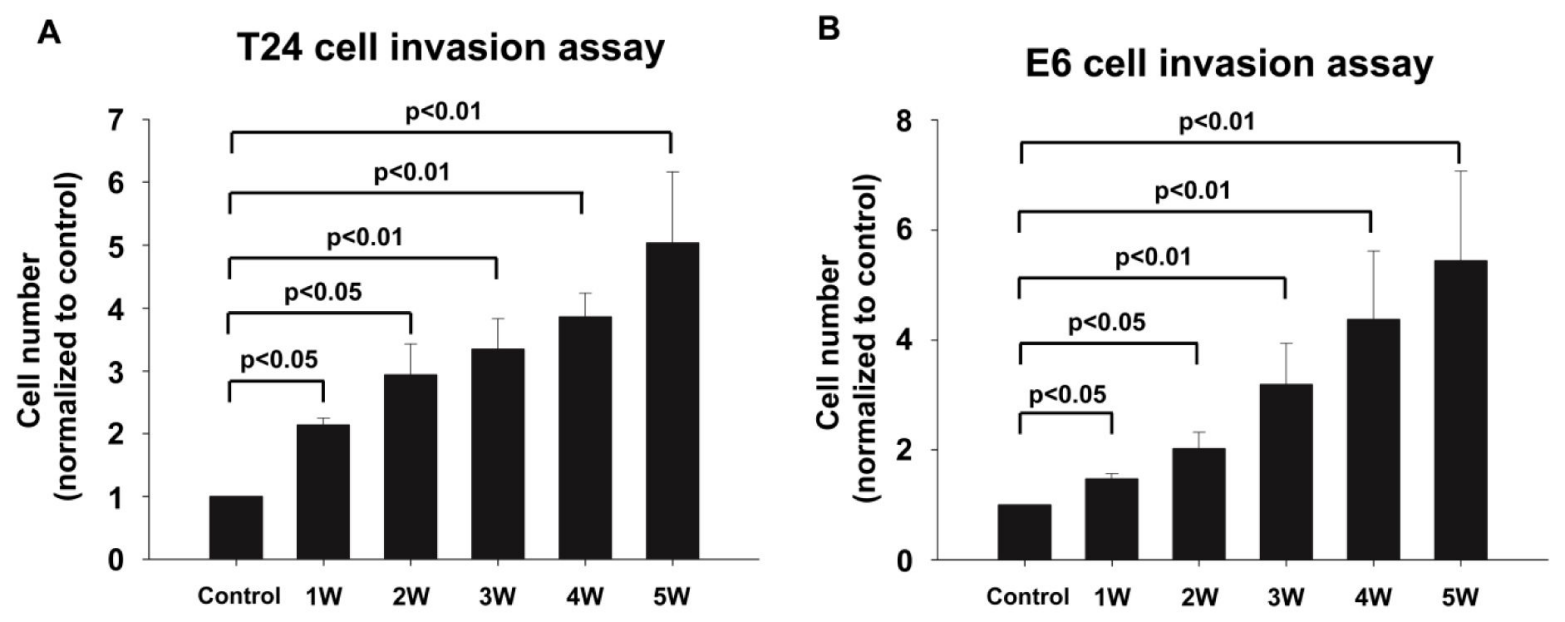
Hydronephrotic urine promoted invasive capability of T24 cells and E6 cells. Hydronephrotic urine from 1 to 5 weeks after UUO promoted the invasion of T24 cells and E6 cells. (A, B) T24 cells and E6 cells were cultured in hydronephrotic urine from 1 to 5 weeks after UUO for 12 hrs and plated in the upper compartment of an 8-µm pore-size transwell invasion chamber which was coated with growth factor-reduced materigel and cultured in serum-free medium for 24 hrs. The numbers of invasive cells were determined by Giemsa staining (Control: without hydronephrotic urine treatment).

The invasive capability of T24 cells and E6 cells was determined by transwell assay, which was coated with growth factor-reduced matrigel. The hydronephrotic urine significantly promoted the invasiveness of T24 cells, increasing the invasive cell numbers by 2.1 fold (UUO-1 weeks), 2.9 fold (UUO-2 weeks), 3.3 fold (UUO-3 weeks), 3.8 fold (UUO-4 weeks) and 5.0 fold (UUO-5 weeks) (Figure 3A). Likewise, the hydronephrotic urine increased the invasive E6 cell numbers by 1.5 fold (UUO-1 weeks), 2.0 fold (UUO-2 weeks), 3.2 fold (UUO-3 weeks), 4.4 fold (UUO-4 weeks) and 5.4 fold (UUO-5 weeks) (Figure 3B).

### Hydronephrotic urine activated the mTORC2-AKT and ERK signaling pathway in normal urothelial cells or urothelial carcinoma cells

**Figure 4:**
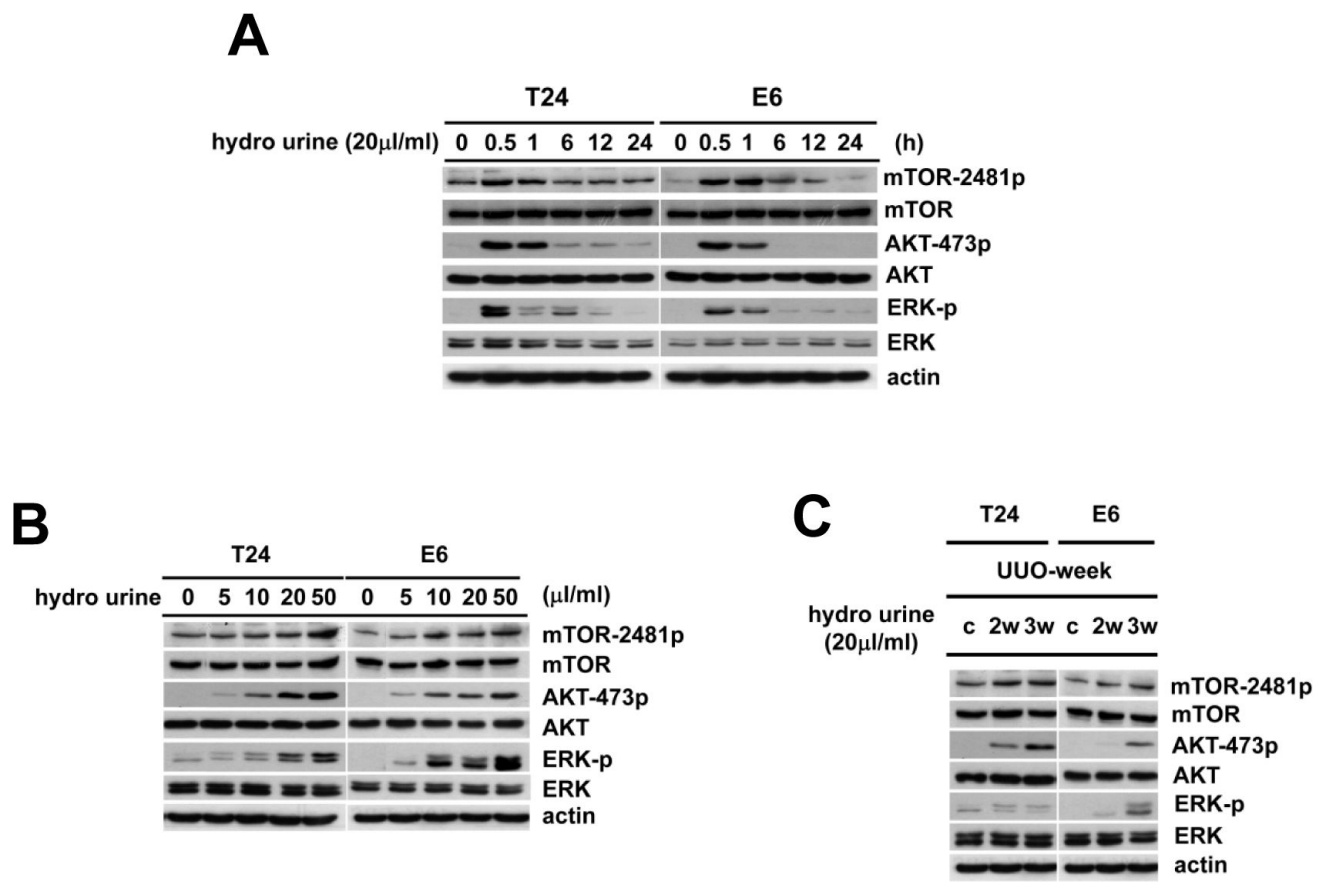
Hydronephrotic urine induced activation of the mTORC2-AKT and ERK pathways in T24 cells and E6 cells. To identify whether ERK and mTORC2-AKT signaling pathway were activated by the hydronephrotic urine in the urothelial carcinoma cell, we analyzed the phosphorylation of mTOR-Ser2481, AKT-Ser473 and ERK in T24 and E6 cells after treatment with hydronephrotic urine. (A) T24 cells and E6 cells were cultured in hydronephrotic urine (20µl/ml) at 3 weeks after UUO for 0, 0.5, 1, 6, 12, 24 hrs, respectively. The phosphorylation of mTOR-Ser2481, AKT-Ser473 and ERK was detected by western blotting. (B) T24 cells and E6 cells were cultured in serum-free medium with 0µl/ml, 5µl/ml, 10µl/ml, 20µl/ml, 50µl/ml hydronephrotic urine from 3 weeks UUO for 30 min, respectively. The phosphorylation of mTOR-Ser2481, AKT-Ser473 and ERK were detected by western blotting. (C) T24 cells and E6 cells were cultured in cultured in serum-free medium with 20µl/ml hydronephrotic urine from 2 and 3 weeks UUO for 30 min. The phosphorylation of mTOR-Ser2481, AKT-Ser473 and ERK by were detected by western blotting (C: Control, without hydronephrotic urine treatment).

**Figure 5:**
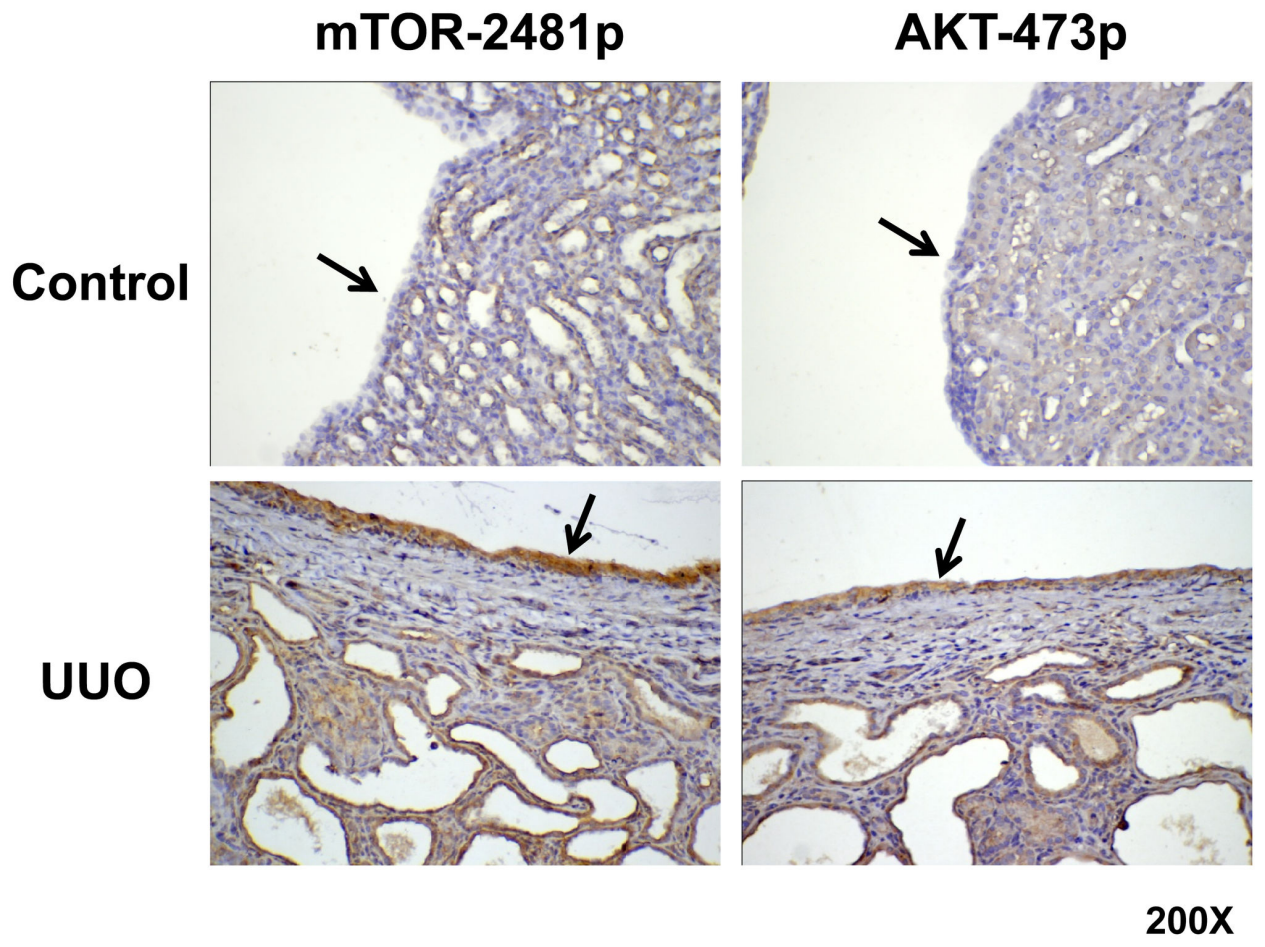
Hydronephrotic urine induced the phosphorylation of mTOR-Ser2481 and AKT-Ser473 in rat kidney after 3 weeks of UUO. The phosphorylation of mTOR-Ser2481 and AKT-Ser473 in normal rat kidney and rat kidney after 3 weeks of UUO was analyzed by immunohistochemistry stain (200X). Arrows indicate the urothelial cell layer in kidney.

To identify whether ERK and mTORC2-AKT signaling were activated by the hydronephrotic urine in the obstructed kidney, we analyzed the phosphorylation of AKT-Ser473, mTOR-Ser2481 and ERK in the T24 cells and E6 cells treated with hydronephrotic urine after 3 weeks of UUO. Figure 4 shows the hydronephrotic urine increased the phosphorylation of mTORC-Ser2481, AKT-Ser473 and ERK in both T24 cells and E6 cells (Figure 4A, B). Figure 4A shows the time course effect during the treatment period. Figure 4B shows that a larger amount of hydronephrotic urine induced more phosphorylation of AKT-Ser473, mTOR-Ser2481 and ERK in both T24 cells and E6 cells. Compared with the hydronephrotic urine treatment after 2 weeks of UUO, the same treatment after 3 weeks UUO induced more phosphorylation of AKT-Ser473, mTOR-Ser2481 and ERK in the T24 cells and E6 cells (Figure 4C). In the immunohistochemistry staining, the phosphorylation of mTOR-Ser2481 and AKT-Ser473 were overexpressed in the urothelial cells of rat kidneys after UUO (Figure 5). These overexpressions were not detected in the control rat kidneys.

### Inhibition of mTORC2-AKT and ERK signaling pathway reduced T24 cells and E6 cells cell viability, migration and invasion in hydronephrotic urine treatment

**Figure 6:**
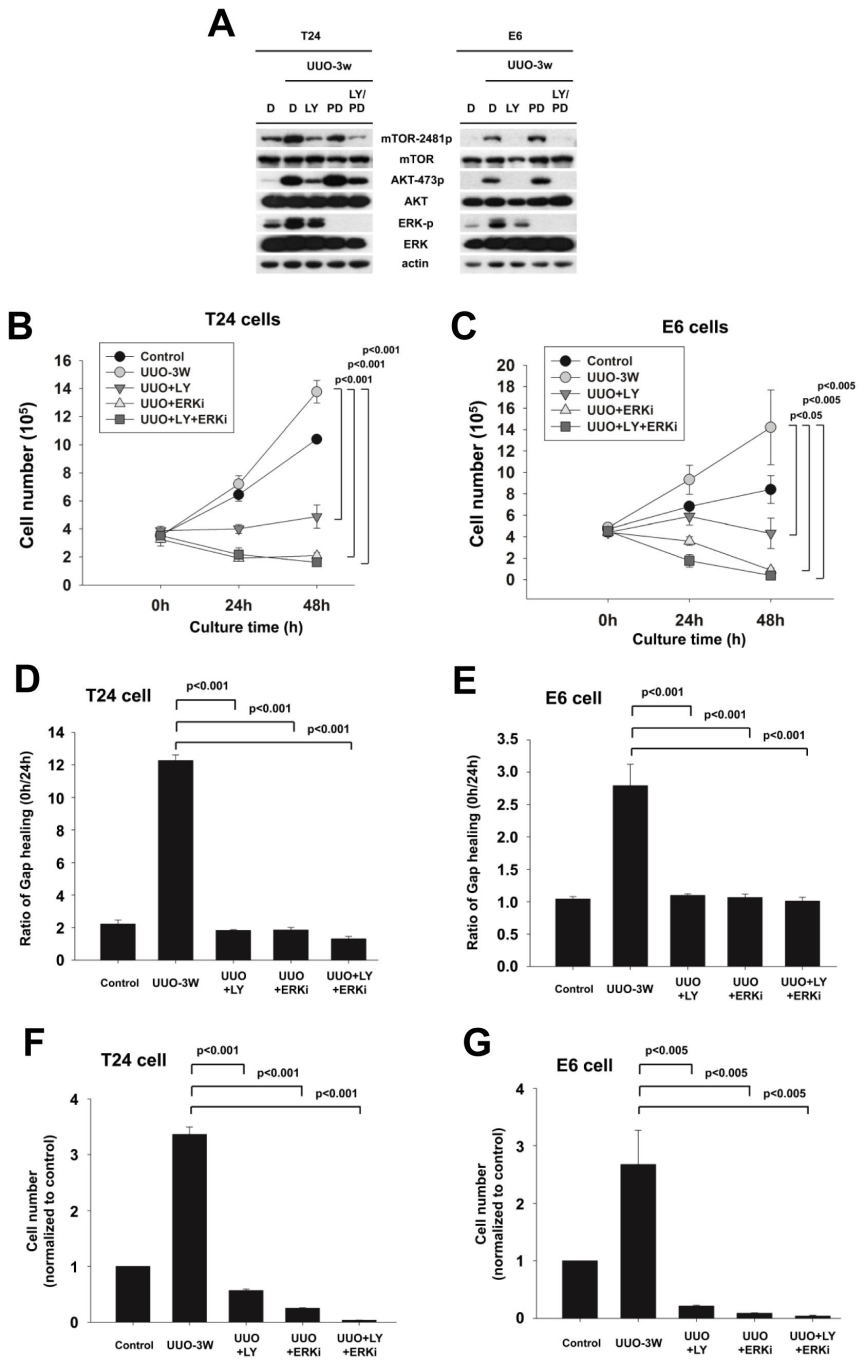
Inhibition of mTORC2-AKT and ERK signaling pathway reduced T24 cells and E6 cells cell viability, migration and invasion in hydronephrotic urine treatment. To confirm the importance of mTORC2-AKT and ERK signaling pathway in T24 and E6 cells after the treatment of hydronephrotic urine, we used LY294002 (PI3K inhibitor) and PD184352 (ERK inhibitor) to analysis the cell function. (A) T24 cells and E6 cells were cultured in serum free medium and treated with LY294002 (20µM) and PD184352 (10µM) for 60 min and stimulated with 20µl/ml hydronephrotic urine from UUO 3 weeks for 30 min. The phosphorylation of mTOR-Ser2481, AKT-Ser473 and ERK were detected by western blotting (D: DMSO, LY: LY294002, PD: PD184352). (B, C) T24 cells and E6 cells were cultured in serum free medium and treated with LY294002 (20µM) and PD184352 (10µM) and stimulated with 20µl/ml hydronephrotic urine from UUO 3 weeks. The cells would be counted the cell number after treatment for 0, 24 and 48 hrs, respectively (Control: without hydronephrotic urine treatment, LY: LY294002, ERKi: PD184352). (D, E) T24 cells and E6 cells were cultured in serum free medium and treated with LY294002 (20µM) and PD184352 (10µM) and stimulated with 20µl/ml hydronephrotic urine from UUO 3 weeks for 24 hrs. The cells would be analyzed the migration capability by wound healing assay (Control: without hydronephrotic urine treatment, LY: LY294002, ERKi: PD184352). (F, G) T24 cells and E6 cells were cultured in serum free medium and treated with LY294002 (20µM) and PD184352 (10µM) and stimulated with 20µl/ml hydronephrotic urine from UUO 3 weeks for 12 hrs. The cells would be analyzed the invasion capability by transwell motility assay (Control: without hydronephrotic urine treatment, LY: LY294002, ERKi: PD184352).

To examine the mechanism of hydronephrotic urine induced activation of mTORC2-AKT and ERK signaling pathway in T24 cells and E6 cells, we used the PI3K inhibitor (LY294002) and ERK inhibitor (PD184352) to inhibit the activity of mTORC2-AKT and ERK in T24 cells and E6 cells. PI3K has been reported to be a critical upstream regulator of mTORC2-AKT signaling pathway [30]. In figure 6A, LY294002 and PD184352 inhibit the phosphorylation of mTOR2481, AKTSer473 and ERK induced by the treatment of hydronephrotic urine in T24 and E6 cells (Figure 6A). These two inhibitors also decreased the levels of cell viability, migration and invasion in T24 and E6 cells (Figure 6B ~ G). Taken together, these findings supported that the mTORC2-AKT and ERK signaling pathway play a critical role in hydronephrotic urine induced urothelial cell carcinoma proliferation, migration and invasion.

### Overexpression of angiogenic growth factors are in the hydronephrotic urine of the obstructed kidney

**Figure 7:**
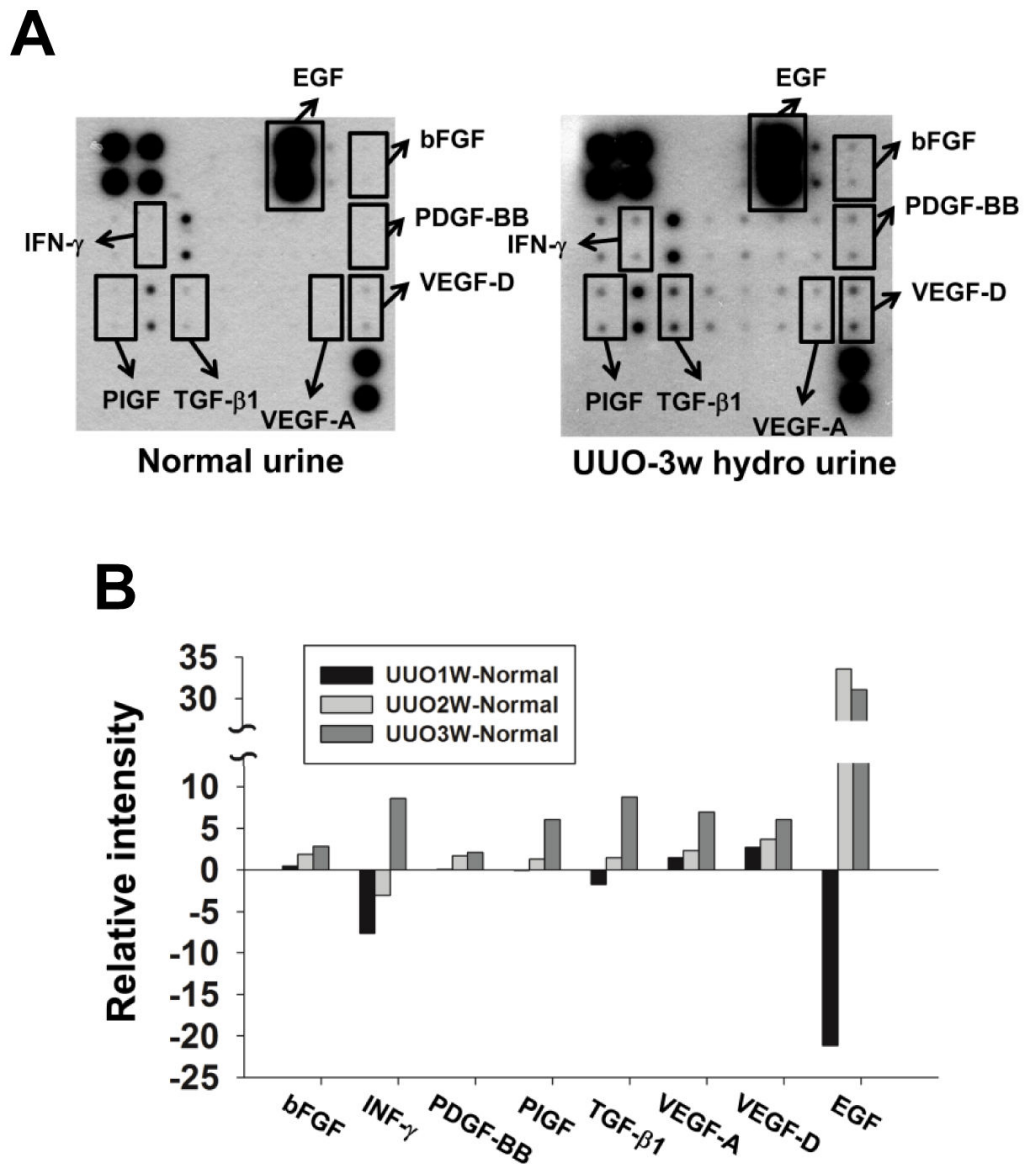
The expression of angiogenetic growth factors and cytokines in the hydronephrotic urine after UUO were higher than those in normal urine. In Human Angiogenesis Antibody Array analysis, the expressions of several growth factors were increased in the hydronephrotic urine from kidneys subjected to unilateral ureteral obstruction. (A) The Human Angiogenesis Antibody Array membrane was incubated with normal rat urine and hydronephrotic urine after 3 weeks UUO for 24 hrs. (B) The expression of bFGF, IFN-γ, PDGF-BB, PIGF, TGF-β, VEGF-A, VEGF-D and EGF in hydronephrotic urine were higher than those in normal urine. The relative intensity indicates the increase and decrease of the intensity of growth factors in hydronephrotic urine minus the intensity of growth factors in normal urine.

The growth factors and cytokines in hydronephrotic urine may promote the migratory and invasive capability of T24 cells and E6 cells. Through the Human Angiogenesis Antibody Array, we found that the expressions of growth factors and cytokines were generally increased in hydronephrotic urine, including basic Fibroblast growth factor (bFGF), interferon-γ (IFN-γ), platelet-derived growth factor-BB (PDGF-BB), placental-derived growth factor (PIGF), transforming growth factor-β (TGF-β), vascular endothelial growth factor-A (VEGF-A), vascular endothelial growth factor-D (VEGF-D) and epidermal growth factor (EGF) (Figure 7A, B).

In both hydronephrotic urine and normal urine, the expression of EGF was much higher than that of the other growth factors. One week after UUO, the EGF decreased significantly but it rapidly increased after 2-3 weeks of UUO. Figure 7B showed the change of relative intensity in the protein array signals which expressed the increase and decrease of the intensity of growth factors and cytokines in hydronephrotic urine minus the intensities of growth factors in normal urine. Although the signaling intensities of IFN-γ, PIGF, TGF-β and EGF were decreased in hydronephrotic urine after 1-2 weeks of UUO, most growth factors and cytokines in hydronephrotic urine were significantly increased with the duration of UUO.

### EGF induces the cell viability, migration, invasion and the activation of mTORC2-AKT and ERK signaling pathway in T24 and E6 cells

**Figure 8:**
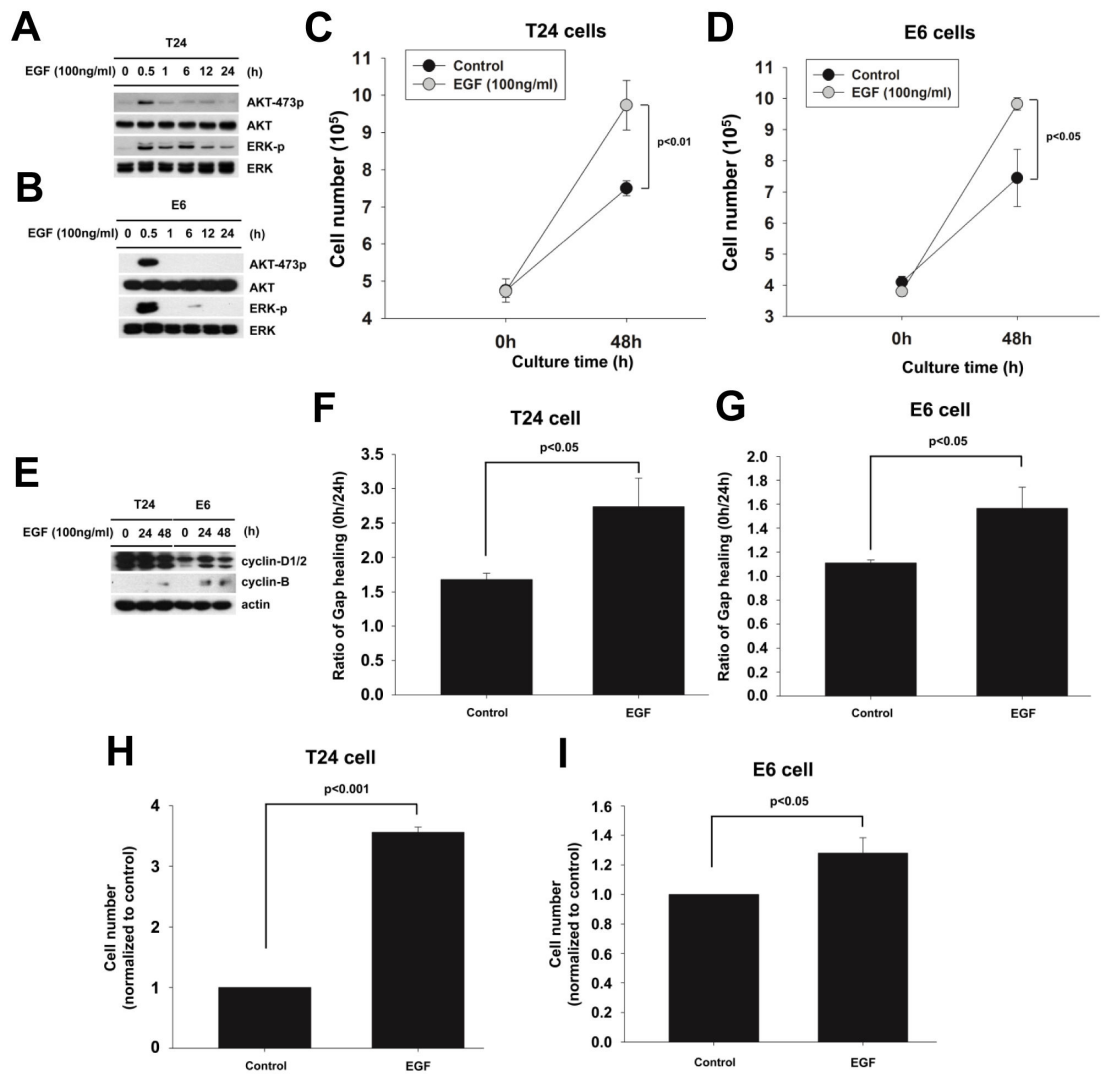
EGF induces proliferation, migration, invasion and activation of mTORC2-AKT and ERK signaling pathway in T24 and E6 cells. To identify whether ERK and mTORC2-AKT signaling pathway were activated by the EGF in the urothelial carcinoma cell, we analyzed the phosphorylation of AKT-Ser473 and ERK in T24 and E6 cells after treatment with EGF. (A, B) T24 cells and E6 cells were cultured in EGF (100ng/ml) for 0, 0.5, 1, 6, 12, 24 hrs, respectively. The phosphorylation of AKT-Ser473 and ERK was detected by western blotting. (C, D) T24 cells and E6 cells were cultured in serum free medium and stimulated with EGF (100ng/ml). The cells would be counted the cell number after treatment for 0 and 48 hrs, respectively. (E) T24 cells and E6 cells were cultured in EGF (100ng/ml) for 0, 24 and 48 hrs, respectively. The expressions of cyclin-B and cyclin-D1/2 were detected by western blotting. (F, G) T24 cells and E6 cells were cultured in serum free medium stimulated with EGF (100ng/ml) for 24 hrs. The cells would be analyzed the migration capability by wound healing assay. (H, I) T24 cells and E6 cells were cultured in serum free medium and stimulated with EGF (100ng/ml) for 12 hrs. The cells would be analyzed the invasion capability by transwell motility assay.

The protein array assay of hydronephrotic urine showed that the expression of EGF is the highest in various growth factors and hormones. The correlation of EGF receptor and high grade urothelial carcinoma had been reported [31]. In this study, EGF really induced mTOR2481, AKTSer473 and ERK phosphorylation (Figure 8A, B). EGF also promoted the T24 and E6 cells proliferation (Figure 8C, D) and increased the protein expression of cyclin-B in T24 and E6 cells and cyclin-D2 in E6 cells (Figure 8E). EGF also promoted more cell migration (Figure 8F, G) and cell invasion (Figure 8H, I). In summary, we demonstrated that similar effects of EGF and hydronephrotic urine to promote urothelial cell carcinoma proliferation, migration and invasion

## Discussion

Urgent relief of chronic hydronephrosis is usually not recommended by urologists because the common complications of hydronephrosis are urinary tract infections and kidney failure, but not the progression of malignancy. However, Urothelial carcinoma is the most common urological cancer in Taiwan [32–34]. Our previous studies and those of other colleagues showed that end-stage renal failure patients and kidney transplant recipients have very high prevalence of urothelial carcinoma in Taiwan [1,2]. The common risk factors for urothelial carcinoma are smoking and exposure to chemical carcinogens [32–34]. Consumption of arsenic-contaminated groundwater and Chinese herbal drugs containing arachidonic acid are additional unique risk factors for urothelial carcinoma in Taiwan [2]. Besides, hydronephrosis is not uncommon in newly-diagnosed urothelial carcinoma in both end-stage renal failure and kidney transplant patients [1,2]. In the past few years, two patients with hydronephrosis eventually develop urothelial carcinoma at our hospital. In fact, no prospective studies have been conducted to evaluate the impact of hydronephrotic urine after ureteral obstruction on the development of urothelial carcinoma.

In this study, our results suggest that hydronephrotic urine itself might promote the urothelial carcinoma progression. The data from our *in vitro* study showed that the hydronephrotic urine from the obstructed kidneys significantly promoted the proliferation, migration and invasion of both urothelial carcinoma (T24) cells and normal urothelial (E6) cells. The activation of cell proliferation was also confirmed by the Western blot analysis of cell cycle proteins. We find that hydronephrotic urine also increases the expression of cyclin-D2 in E6 cells, cyclin-B and CDK2 in T24 cells and E6 cells and decreases the expression of p27 and p21 in these two cell types. Moreover, the cellular activation by hydronephrotic urine depends on the mTORC2-AKT and ERK signaling pathways which have been shown to be upregulated in the cancer cells.

The growth factors dependent activation of ERK and mTORC2-AKT signaling pathway play an important role in the pathogenesis of human cancers [24–26]. Increased activity of these signaling pathway has also been reported in urothelial carcinoma [35]. In our study, we suggest that hydronephrotic urine may induce activation of these two signaling pathway and may promote urothelial carcinoma cell proliferation, migration and invasion.

Higher expression levels of PDGF, TGF-β, IGF-I, IL-6 have been reported after ureteral obstruction [7,8,10,11]. In agreement with the previous studies, our study demonstrates that growth factors and cytokines, including bFGF, IFN-γ, PDGF-BB, PIGF, TGF-β, VEGF-A, VEGF-D and EGF, were overexpressed in the hydronephrotic urine samples collected after 1-5 weeks of UUO. The decrease of INF-γ, TGF-β and EGF in the first week after UUO may be related to the effect of acute tubular injury. However, all the growth factors we detected by array assay were overexpressed on a time-dependent manner. Importantly, EGF plays a crucial role in urothelial carcinoma. In one study a high ratio of positive EGF receptor was found in 50% high grade urothelial carcinoma [31]. The increase of these growth factors should be considered as the provocative factors for urothelial carcinoma. Treatment options include aggressive relief of hydronephrosis or preventive nephrectomy for the obstructive non-functional kidney, especially in patients with higher risk to develop urothelial carcinoma, such as patients under regular dialysis and kidney transplant recipients in Taiwan.

Recent clinical reports indicate that the higher grade of preoperative hydronephrosis may be associated with the poorer pathological outcomes in patients undergoing nephroureterectomy for urothelial carcinoma [4]. Hoshino and colleagues also reported that the grade of hydronephrosis may be helpful in the clinical decision-making for adjuvant chemotherapy [36]. Furthermore, Ng et al. demonstrated that hydronephrosis was associated with features of aggressive disease and predictive of advanced pathologic stage for upper-tract urothelial carcinoma [37]. They suggest that hydronephrosis can be a valuable prognostic tool for preoperative planning and counseling regarding disease outcomes. Taken together, the clinical association between hydronephrosis and the outcome of urothelial carcinoma support the hypothesis that hydronephrosis may exacerbate the progression of urothelial carcinoma.

However, our study does have some limitations. Particular challenging is the effect seen may not be specific to obstruction. One might hypothesize that other causes of renal inflammation might result in the release of stimulatory or pro-invasive factors. Additional experiments and additional controls would be needed to address this possibility.

In summary, having acknowledged the limitation of this study, we can nevertheless confirm that hydronephrotic urine promote the progression of urothelial carcinoma through the activation of the mTORC2-AKT and ERK signaling pathways. These results may suggest the aggressive relief of hydronephrosis or preventive nephrectomy for the obstructive non-functional kidney in the endemic area of urothelial carcinoma. Further investigation using live animal models of tumor growth may be needed to clarify aspects of these statements.

## Supporting Information

Figure S1 **The effects on T24 cells and E6 cells cell functions and mTORC2-AKT and ERK signaling pathway in normal urine treatment.** To identify whether ERK and mTORC2-AKT signaling pathway were activated by the normal urine in the urothelial carcinoma cell, we analyzed the phosphorylation of mTOR-Ser2481, AKT-Ser473 and ERK in T24 and E6 cells after treatment with normal urine. (A) T24 cells and E6 cells were cultured in normal urine (20µl/ml) for 30 min. The phosphorylation of mTOR-Ser2481, AKT-Ser473 and ERK was detected by western blotting (Control: without hydronephrotic urine treatment, NU: normal urine). (B, C) T24 cells and E6 cells were cultured in serum free medium and stimulated with normal urine (20µl/ml). The cells would be counted the cell number after treatment for 0, 24 and 48 hrs, respectively (Control: without hydronephrotic urine treatment). (D) T24 cells and E6 cells were cultured in normal urine (20µl/ml) for 0, 24 and 48 hrs, respectively. The expression of cyclin-B and cyclin-D1/2 was detected by western blotting. (E, F) T24 cells and E6 cells were cultured in serum free medium stimulated with normal urine (20µl/ml) for 24 hrs. The cells would be analyzed the migration capability by wound healing assay (Control: without hydronephrotic urine treatment). (G, H) T24 cells and E6 cells were cultured in serum free medium and stimulated with normal urine (20µl/ml) for 12 hrs. The cells would be analyzed the invasion capability by transwell motility assay (Control: without hydronephrotic urine treatment).
(TIF)
