# Correction: Hydronephrotic Urine in the Obstructed Kidney Promotes Urothelial Carcinoma Cell Proliferation, Migration, Invasion through the Activation of mTORC2-AKT and ERK Signaling Pathways

**DOI:** 10.1371/journal.pone.0142702

**Published:** 2015-11-05

**Authors:** Chi-Hao Chang, Jian-Ri Li, Kuo-Hsiung Shu, Yun-Ching Fu, Ming-Ju Wu

Errors were introduced to Fig 4 during the manuscript preparation process. Specifically, in Fig 4A the mTOR panel from the T24 cells appears to be identical to the mTOR panel from the E6 cells and the actin panel from the T24 cell also appears to be identical to the actin panel from the E6 cells. The authors have provided the correct version of Fig 4 here.

**Fig 4:**
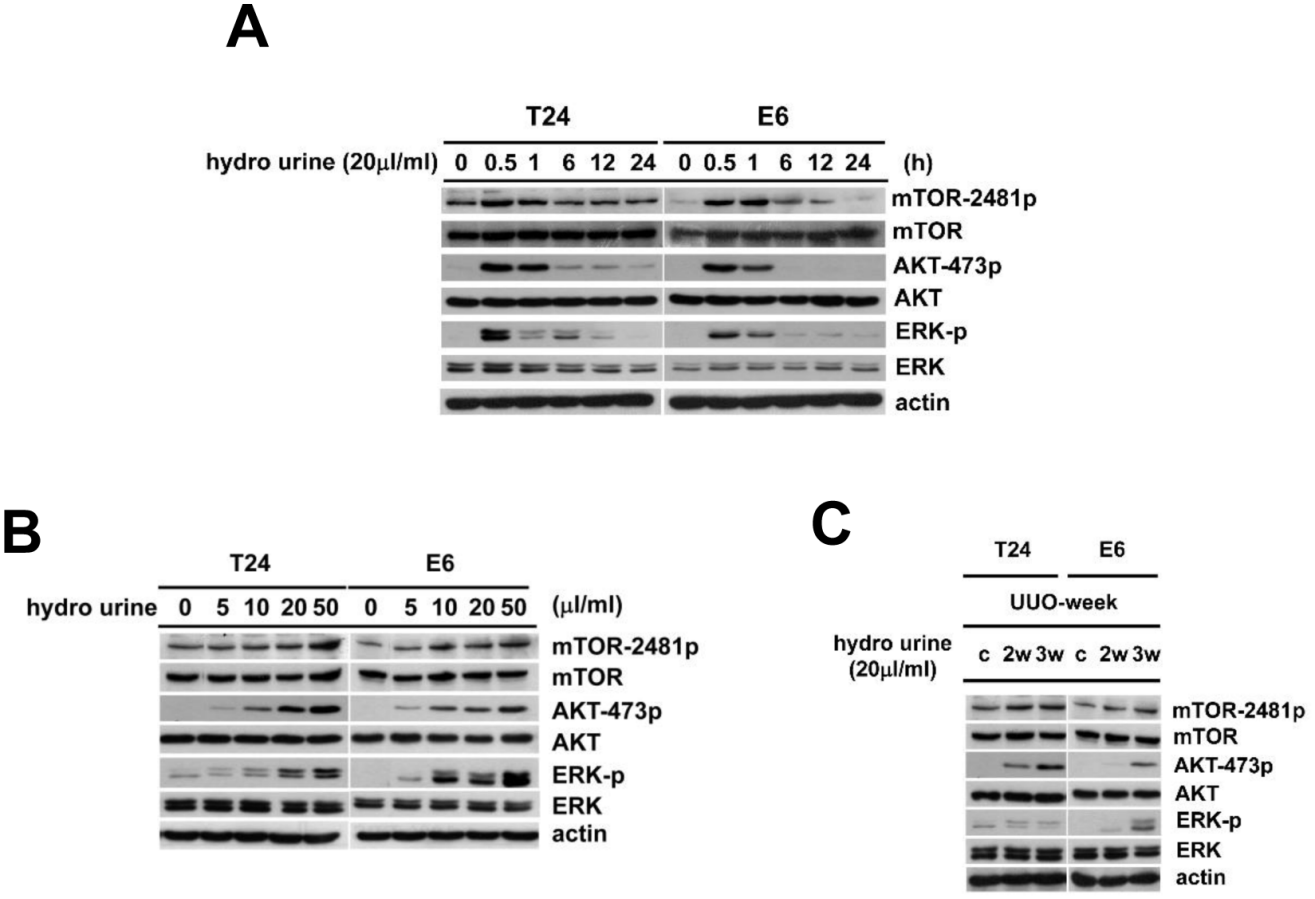
Hydronephrotic urine induced activation of the mTORC2-AKT and ERK pathways in T24 cells and E6 cells. To identify whether ERK and mTORC2-AKT signaling pathway were activated by the hydronephrotic urine in the urothelial carcinoma cell, we analyzed the phosphorylation of mTOR-Ser2481, AKT-Ser473 and ERK in T24 and E6 cells after treatment with hydronephrotic urine. (A) T24 cells and E6 cells were cultured in hydronephrotic urine (20μl/ml) at 3 weeks after UUO for 0, 0.5, 1, 6, 12, 24 hrs, respectively. The phosphorylation of mTOR-Ser2481, AKT-Ser473 and ERK was detected by western blotting. (B) T24 cells and E6 cells were cultured in serum-free medium with 0μl/ml, 5μl/ml, 10μl/ml, 20μl/ml, 50μl/ml hydronephrotic urine from 3 weeks UUO for 30 min, respectively. The phosphorylation of mTOR-Ser2481, AKT-Ser473 and ERK were detected by western blotting. (C) T24 cells and E6 cells were cultured in cultured in serum-free medium with 20μl/ml hydronephrotic urine from 2 and 3 weeks UUO for 30 min. The phosphorylation of mTOR-Ser2481, AKT-Ser473 and ERK by were detected by western blotting (C: Control, without hydronephrotic urine treatment).

The authors confirm that these changes do not alter their findings. The authors have provided raw, uncropped blots as Supporting Information.

## Supporting Information

S1 File Uncropped Blots. (PPTX)
