# Supplementary figures and images for: Hydronephrotic Urine in the Obstructed Kidney Promotes Urothelial Carcinoma Cell Proliferation, Migration, Invasion through the Activation of mTORC2-AKT and ERK Signaling Pathways
Source: PLoS One. 2013 Sep 4;8(9):e74300. doi: 10.1371/journal.pone.0074300 (PMC3762757; doi:10.1371/journal.pone.0074300)

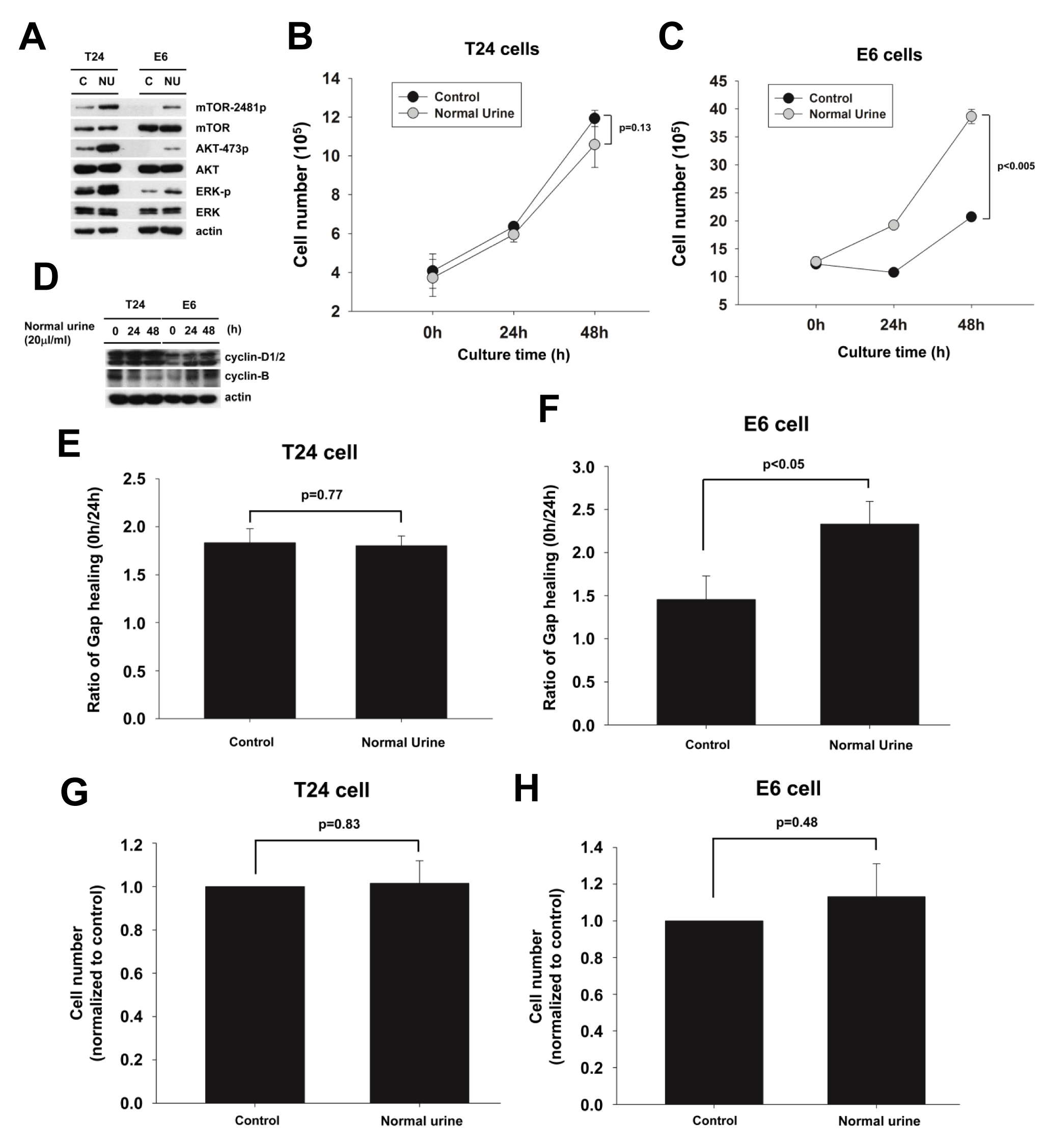

Supplement: Figure S1 — The effects on T24 cells and E6 cells cell functions and mTORC2-AKT and ERK signaling pathway in normal urine treatment. To identify whether ERK and mTORC2-AKT signaling pathway were activated by the normal urine in the urothelial carcinoma cell, we analyzed the phosphorylation of mTOR-Ser2481, AKT-Ser473 and ERK in T24 and E6 cells after treatment with normal urine. (A) T24 cells and E6 cells were cultured in normal urine (20µl/ml) for 30 min. The phosphorylation of mTOR-Ser2481, AKT-Ser473 and ERK was detected by western blotting (Control: without hydronephrotic urine treatment, NU: normal urine). (B, C) T24 cells and E6 cells were cultured in serum free medium and stimulated with normal urine (20µl/ml). The cells would be counted the cell number after treatment for 0, 24 and 48 hrs, respectively (Control: without hydronephrotic urine treatment). (D) T24 cells and E6 cells were cultured in normal urine (20µl/ml) for 0, 24 and 48 hrs, respectively. The expression of cyclin-B and cyclin-D1/2 was detected by western blotting. (E, F) T24 cells and E6 cells were cultured in serum free medium stimulated with normal urine (20µl/ml) for 24 hrs. The cells would be analyzed the migration capability by wound healing assay (Control: without hydronephrotic urine treatment). (G, H) T24 cells and E6 cells were cultured in serum free medium and stimulated with normal urine (20µl/ml) for 12 hrs. The cells would be analyzed the invasion capability by transwell motility assay (Control: without hydronephrotic urine treatment). (TIF) [file pone.0074300.s001.tif]
