# Supplementary figures and images for: Correction: Hydronephrotic Urine in the Obstructed Kidney Promotes Urothelial Carcinoma Cell Proliferation, Migration, Invasion through the Activation of mTORC2-AKT and ERK Signaling Pathways
Source: PLoS One. 2015 Nov 5;10(11):e0142702. doi: 10.1371/journal.pone.0142702 (PMC4634949; doi:10.1371/journal.pone.0142702)

## Slide 1
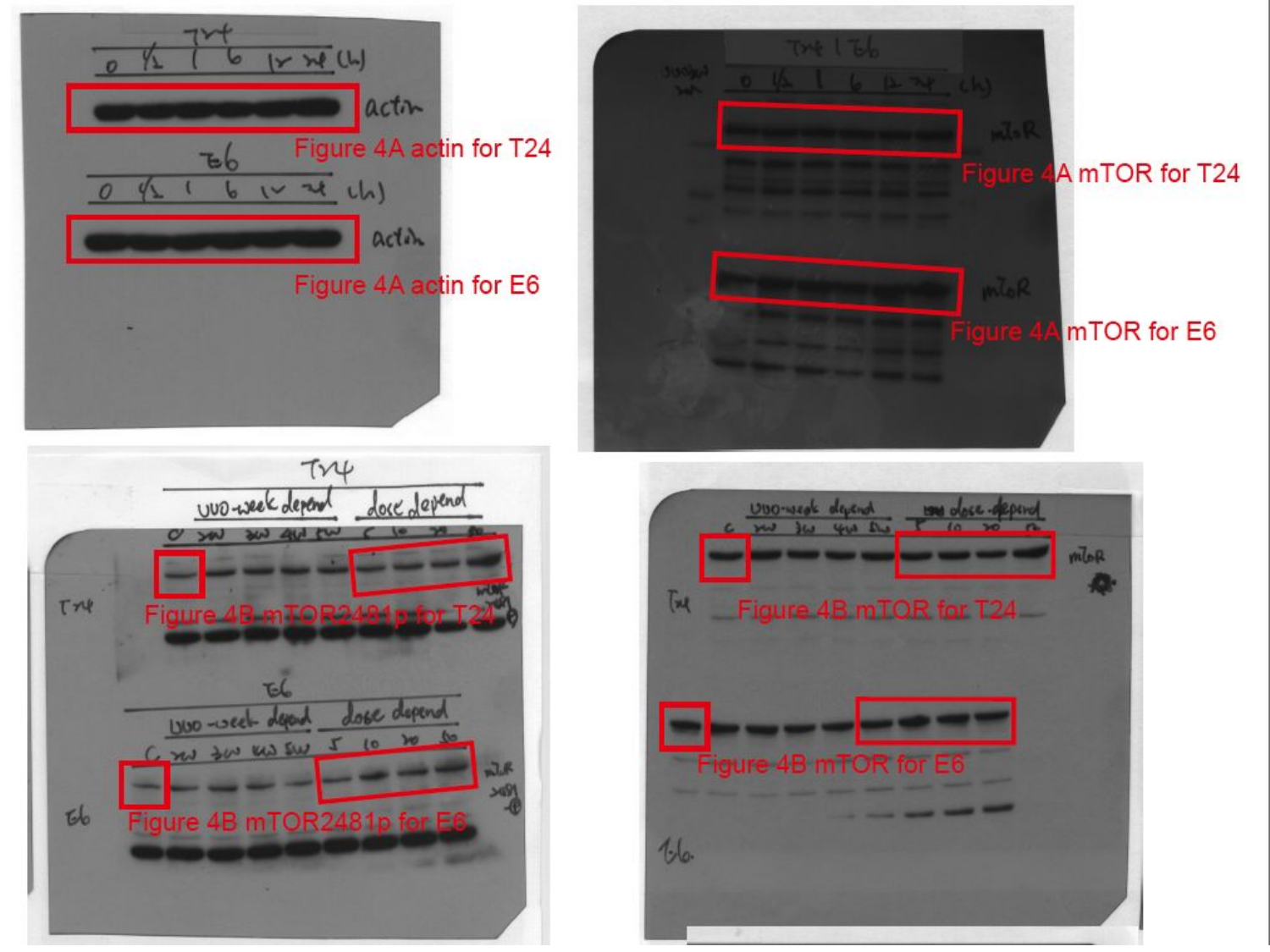

## Slide 2
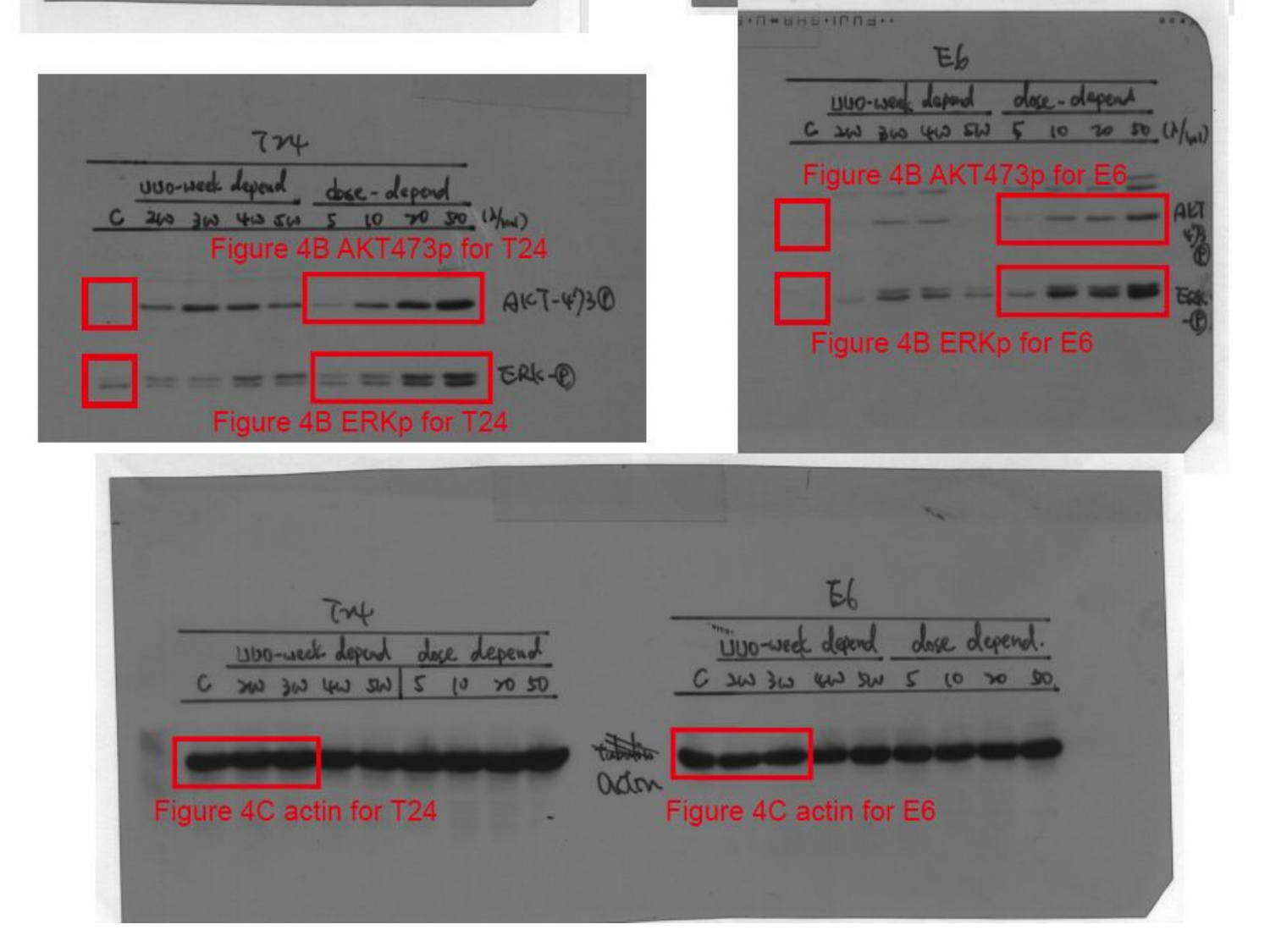

Supplement: S1 File — (PPTX) [file pone.0142702.s001.pptx]
